# The clinical utility of circulating human papillomavirus across squamous cell carcinomas

**DOI:** 10.2340/1651-226X.2025.41288

**Published:** 2025-01-02

**Authors:** Karen-Lise G. Spindler, Anne V. Jakobsen, Jesper G. Eriksen, Lars Fokdal, Marianne Nordsmark, Lise B.J. Thorsen, Karen L. Wind, Anna C. Lefevre, Jens Overgaard

**Affiliations:** a Department of Experimental Clinical Oncology, Aarhus University Hospital, Denmark; b Department of Clinical Medicine, Aarhus University, Denmark; c Department of Oncology, Vejle Hospital, Denmark

**Keywords:** Squamous cell carcinoma, circulating tumor DNA (ctDNA), Human papillomavirus

## Abstract

**Background and purpose:**

The similarities in biology, treatment regimens and outcome between the different human papillomavirus (HPV) associated squamous cell carcinomas (SCCs) allow for extrapolation of results generated from one SC tumor type to another.

In HPV associated cancers, HPV is integrated into the tumor genome and can consequently be detected in the circulating fragments of the tumor DNA. Thus, measurement of HPV in the plasma is a surrogate for circulating tumor DNA (ctDNA) and holds promise as a clinically relevant biomarker in HPV associated cancers.

With the present overview we aim to present the status of circulating HPV studies in SCCs, the clinical potential and the gaps of knowledge, with the overall aim to facilitate the next steps into clinically relevant prospective trials.

**Material and methods:**

We reviewed the literature and presented the data for each tumor type as well as analyses of the clinical utility across the SCC.

**Results and interpretation:**

A total of 41 studies were identified in cervical, head and neck and anal SCC and we discuss the common signals from the results across the different tumor sites. Our results not only confirm the strong clinical potential but also emphasize an urgent need to coordinate studies to allow for relevant sample sizes and statistical validations.

## Introduction to the biology of Squamous Cell Carcinomas

Squamous cell carcinoma is a frequent cancer type that develops from the squamous epithelium, which typically regenerates within 2–3 weeks and withstands external trauma and abrasions and has an ability to accelerate its repopulation to replace traumatized areas. Human papilloma virus (HPV) is a dominating and increasing etiological factor [1]. HPV-positive squamous cell carcinomas (SCCs) are frequently found in the uterine cervix (> 95%), head and neck region, especially the oropharynx (40–80%), upper part of the esophagus (20%), vulva (50%) and anal canal (> 80%).

All SCCs exhibit a characteristic pattern of behavior and response to treatment. The diseases are primarily loco-regional and the primary treatment is surgery and/or (chemo)radiotherapy (CRT) [2–4]. SCCs are characterized by showing a steep dose-response relationship and accelerated radiotherapy regimens can be relevant [5, 6]. Hypoxia, the EGFR biology and the immune environment are important common biological features [2, 7–14].

### Human papillomavirus in squamous cell carcinomas

Human papillomavirus status plays a role in sensitivity to both radiotherapy and systemic treatment, but results are contradictory. The HPV relation seems to influence radiosensitivity in some (i.e. the oropharynx) tumors more than others [9, 15, 16]. The mechanism and the biological differences between HPV-positive and HPV-negative disease is unknown and they are currently treated with the same regimens and radiation doses.

There are several different subtypes of HPV, which can be classified into high-risk (oncogenic) and low-risk subtypes, but we have little knowledge of the role of the different HPV subtypes in relation to treatment sensitivity and outcome [16–19].

### Pan SCC features

Most SCCs are sensitive to radiotherapy, platinum-based chemotherapy and, as recently reported, checkpoint inhibition, but biological features and predictive markers for response to these different treatment modalities are inadequately characterized. When treating primary, localized SCC with CRT, identification of biomarkers to guide personalized treatment is crucial to improve selection of ‘poor responders’ who may benefit from treatment intensification for a curative approach despite the risk of increasing toxicity, and to identify ‘good responders’, where decreasing radiation doses and potential side effects may be possible.

The optimal time-point for final assessment of complete response (CR) after CRT is not identified and with current practice, premature decisions may lead to unnecessary salvage surgery. In case of contradicting imaging and biopsy results, predictive markers could aid in the decision for salvage surgery. Finally, there is an urgent need for tools for early detection of recurrences with the possibility of cure through localized treatment.

Palliative chemotherapy for SSCs implies a high risk of side effects and limited benefit in some patients. In the palliative setting predictive markers will enable more rapid change of ineffective systemic treatment strategies. Pre-clinical data support that HPV-positive tumors are more susceptible to immunotherapy with checkpoint inhibitors, but optimal selection for therapy is warranted [20].

### Circulating HPV as circulating tumor DNA measurement

**Figure 1:**
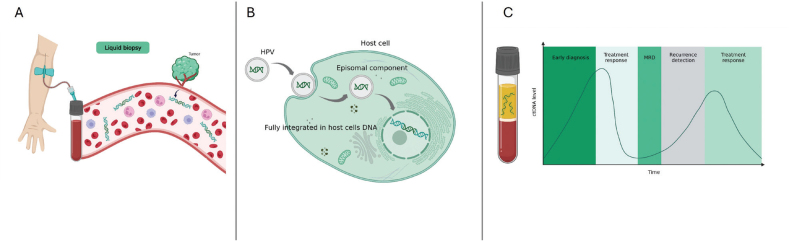
(A) Liquid biopsy. Circulating tumor DNA (ctDNA) represents small fragments of DNA from tumor cells that are released into the bloodstream and can be detected in a simple blood sample. In HPV associated cancers, HPV DNA can be detected in the circulating tumor DNA fragments. (B) HPV integration. The integration of HPV into the host cell can be full integration with the virus DNA being spliced into the host cell’s DNA or by partial or full episomal integration. Notably, recent data have suggested that measures of HPV integration in itself have a biological and prognostic impact [72]. (C) ctDNA levels during the disease and treatment course. The elimination half-life of ctDNA is less than a few hours, leading to rapid biological clearance from the bloodstream following the curative removal of tumor tissue. The presence of ctDNA in plasma following treatment indicates microscopic residual disease (MRD) and is generally associated with a poor outcome. Created with BioRender.com (Jakobsen, A. (2025) https://BioRender.com/w47u880)

Circulating tumor DNA (ctDNA) represents small DNA fragments with tumor specific characteristics and can be detected in a simple blood sample (Figure 1). This has gained considerable interest as a prognostic and predictive marker in both localized and metastatic cancer disease. The elimination half-life of ctDNA is only a few hours and biological clearance from the bloodstream is consequently expected immediately following curative removal of tumor tissue [21]. It is now established that the presence of ctDNA in plasma post-surgery indicates microscopic residual disease (MRD) and a subsequent very poor outcome [22, 23]. Fundamental aspects of detection and measurement of ctDNA comprises (1) biological knowledge of the individual tumor type (is the tumor cells likely to shed ctDNA or not? are there easily measurable and frequent well known tumor specific mutations or epigenetic alterations to measure?), (2) the laboratory methods (is a broad method that targets many different alterations necessary or can testing be covered by simple methods on few targets?) and (3) clinical need (is an ultra-high sensitivity or specificity needed? is it necessary to use a tumor informed approach or can a pragmatic tumor agnostic strategy be used?).

In HPV associated cancers, HPV is integrated into the tumor genome (Figure 1) and can be detected in the circulating fragments of the tumor DNA [24]. Thus, measurement of HPV in the plasma is a surrogate for ctDNA and holds promise as a clinically relevant biomarker in HPV associated cancers.

The shared characteristics in biology, treatment approaches, and outcomes among HPV-associated SCCs allow for extrapolation of results from one squamous cell tumor type to another. This overview aims to present the current status of HPVctDNA research in SCCs, highlighting clinical potential and the gaps of knowledge.

## Results

### HPVctDNA in squamous cell carcinoma of the Anus

Nine studies were identified (2 case reports), comprising a total of less than 300 patients, most in the primary setting, and 3 in metastatic disease (Table 1). A study of HPV associated cancer included 15 patients with SCCA. HPVctDNA was present in 87–93% of HPV-positive tumors, in microinvasive carcinomas, but not in blood samples from patients with HPV associated high grade neoplasia [24]. Another study in SCCA showed that HPVctDNA could be detected before CRT in 29 of 33 patients with stage II-III disease, that levels dropped markedly during CRT, and further, that residual detectable HPVctDNA after CRT (3/18 patients) was strongly associated with shorter disease-free survival [25]. Bernard-Tessier et al. demonstrated that HPVctDNA was associated with prognosis during first line chemotherapy in advanced SCCA [26]. Recently, analysis in 88 SCCA patients revealed that pre-treatment level of HPVctDNA was associated with clinical stage and prognosis [19]. Furthermore, three distinct patterns of HPVctDNA elimination during CRT were observed with significantly different risks of local or distant failures. HPVctDNA measurements during FU indicated a strong potential for the prediction of recurrences with a clinically relevant lead time as marker of MRD (Table 2) [19, 27].

**Table 1:** An overview of published studies on HPVctDNA including information on methods, pre-treatment characteristics and prognostic value.

Reference	Tumor site	Sample size	Stages included	Method	Material	Detected HPV subtypes	Concordance to tissue HPV status/p16	Correlation to stage	Prognostic value
**Cervical cancer**									
Kedzia et al. 1992 [39]	CSCC CS	5 4	Stage I-III	Southern blot hybridization	Whole blood	16	Sensitivity 80%	ND	ND
Pornthanakasem et al. 2001 [41]	CSCC Healthy	63 20	Stages I-IV	PCR	Plasma	16, 18	Sensitivity 12%	+	Statistical tendency
Liu et al. 2001 [47]	CSCC	60	Stages I-IV	PCR	Serum	16, 18	Sensitivity 20%	ND	ND
Dong et al. 2002 [48]	CSCC CS Healthy	175 57 60	Stages I-III(IV)	qPCR	Plasma	16, 18	Sensitivity 18% 66% samples collected post-treatment	(+)	ND
Sathish et al. 2004 [49]	CSCC CS Healthy	58 10 30	Stages I-IV	PCR	Plasma	16, 58	Sensitivity 15%	(+)	ND
Kay et al. 2005 [50]	CSCC CS Healthy	45 32 77	Stage I-IV	PCR	Whole blood	16, 18	Sensitivity 24%	(+)	ND
Shimada et al. 2010 [51]	CSCC CS Healthy	20 22+3 20	Stage I-IV	qPCR	Plasma	16	Sensitivity 30%	ND	ND
Campitelli et al. 2012 [44]	CSCC	16	Stage I-IV	qPCR	Serum	16, 18	Sensitivity 81%	(+)	ND
Kang et al. 2017 [52]	CSCC Healthy	21 45	M+	ddPCR	Serum	16, 18	Sensitivity 100%	ND	ND
Cheung et al. 2019 [42]	CSCC	138	Stage I-IV	ddPCR	Plasma	16, 18	Sensitivity 62%	(+)	Statistical tendency
Cabel et al. 2021 [40]	CC	55	Stage I-IV	ddPCR	Serum/plasma	16, 18, 31, 33, 35, 45, 52, 58, 73	Sensitivity 69%	+	No prognostic value
Jeannot et al. 2021 [43]	CC	94	Stage I-IV	ddPCR	Serum	16, 18	Sensitivity 63 %	+	No prognostic value
Bønløkke et al. 2022 [53]	CC CS Healthy	60 8 15	Stage I-IV	ddPCR	Plasma	16, 18	Sensitivity 37%	+	ND
**Head and neck cancer**									
Cao et al. 2012 [37]	OPSCC Healthy	64 10	Stage I-IV	qPCR	Plasma	16, 18	Sensitivity 65%	+	ND
Ahn et al. 2014 [54]	HNSCC	93	Stage I-IV	qPCR	Plasma Saliva	16	Sensitivity 67%	ND	ND
Wang et al. 2015 [55]	HNSCC	47	Stage I-IV	ddPCR	Plasma Saliva	16	Sensitivity 86%	(+)	ND
Dahlstrom et al. 2015 [30]	OPSCC	262	Stage I-IV	qPCR	Serum	16	Sensitivity 60%	+	No prognostic value
Mazurek et al. 2016 [56]	OPSCC Healthy	200 15	Stage I-IV	PCR	Plasma	16, 18	Sensitivity 14%	ND	ND
Lee et al. 2017 [29]	HNSCC	88 (test validation cohorts)	Stage III-IV	NGS	Plasma	16	Sensitivity 90–100%	÷	ND
Hanna et al. 2018 [31]	OPSCC	22	Stage IV or M+	ddPCR	Plasma	16, 18, 31, 33, 45 (investigated)	Sensitivity 71%	+	Prognostic value
Hanna et al. 2019 [32]	OPSCC	21	Stage IV or M+	ddPCR	Plasma	16, 18, 31, 33, 45 (investigated)	Sensitivity 76 %	+	Plasma ctHPV correlated with prognostic score. Salivary did not.
Chera et al. 2019 [33]	HNSCC Controls	103 115	Stage I-IV	ddPCR	Plasma	16, 31, 33, 35	Sensitivity 89%	+	ND
Chera et al. 2020 [38]	HNSCC	115*	Stage I-III	ddPCR	Plasma	16, 18, 31, 33, 35 (investigated)	Sensitivity 99%	ND	ND
Rutkowski et al. 2020 [57]	OPSCC	216	Stage I-IV	PCR	Plasma	16	ND	+	ND
Reder et al. 2020 [58]	OPSCC	50	Stage I-IV or M+	qPCR	Plasma	16	Sensitivity 87 %	+	ND
Tanaka et al. 2021 [28]	HNSCC	35	Stage II-IV	ddPCR	Plasma	16	Sensitivity 100%	ND	ND
Haring et al 2021 [59]	OPSCC	16	R/M+	ddPCR	Plasma	16, 18	Sensitivity 75%	ND	ND
Siravegna et al. 2022 [60]	HNSCC Healthy	70 70	Stage I-IV	ddPCR	Plasma	16, 35, 45	Sensitivity 98%	+	ND
Tanaka et al. 2022 [61]	HNSCC	35	Stage II-IV	ddPCR	Plasma	16	Sensitivity 100%	+	No correlation to failure.
Routman et al. 2022 [62]	OPSCC	45	Stage I-IV(III?)	ddPCR	Serum	16, 18, 33, 35	Sensitivity 89% (76–96%)	ND	ND
Cao et al. 2022 [34]	OPSCC	34	Stage III	ddPCR	Plasma	16, 18	Sensitivity 82%	+	Higher levels were associated with progres-sion within 12 months
Adrian et al. 2023 [35]	OPSCC	136	Stage I-IV	qPCR Luminex multiplex	Plasma	16 +9 subtypes	Sensitivity 79%	+	Prognostic value
Califano et al. 2023 [36]	OPSCC	233	Stage III-IV	qPCR	Plasma Saliva	16	Sensitivity 75–87%	ND	No prognostic value
**Anal cancer**									
Cabel et al. 2017 [63]	SCCA	1	M+	ddPCR	Plasma	16	ND	ND	ND
Cabel et al. 2018 [25]	SCCA	33	Stage II-III	ddPCR	Serum/plasma	16, 18	Sensitivity 88 %	+	No prognostic value
Bernard-Tessier et al. 2019 [26]	SCCA	59	Stage IV or M+	ddPCR	Serum	16	Sensitivity 91 %	÷	Prognostic value
Veyer et al. 2019 [64]	SCCA	1	M+	ddPCR	Plasma	16	ND	ND	ND
Lee et al. 2020 [65]	SCCA	21	Stage I-III	NGS	Plasma	16, 18, 31, 33, 35, 45, 52, 58 (investigated)	Sensitivity 100 %	÷	ND
Lefevre et al. 2021 [19]	SCCA	88	Stage I-IV	ddPCR	Plasma	18, 31, 33, 51, 58	Sensitivity 82 %	+	Statistical tendency
Mazurek et al. 2023 [27]	SCCA	62	Stage I-IV	qPCR	Plasma	16	Sensitivity 87%	+	Prognostic value
**Mixed tumor sites**									
Jeannot et al. 2016 [24]	OPSCC CSCC SCCA	8 47 15	Stage I-IV	qPCR ddPCR	Serum	16, 18	Sensitivity 87%	+	ND
Damerla et al. 2019 [66]	HNSCC SCCA Healthy	97 8 27	Stage I-IV	PCR	Plasma	16, 33	Sensitivity 96%	+	ND

HPV: human papilloma virus. HPVctDNA: circulating human papilloma virus DNA. CSCC: cervical squamous cell carcinoma. CS: cervical carcinoma in situ. HNSCC: head and neck squamous cell carcinoma. OPSCC: oropharyngeal squamous cell carcinomas. SCCA: squamous cell carcinoma of the anus. M+: metastatic disease. R: recurrent disease. PCR: polymerase chain reaction. qPCR: quantitative polymerase chain reaction. ddPCR: droplet digital polymerase chain reaction. NGS: next generation sequencing. ND: Not done.

* 115 included with post-treatment samples, 86 had pre-treatment samples available.

**Table 2:** An overview of publications with repeated measurements of HPVctDNA.

Reference	Tumor site	Sample size total	Sample size Pre-treatment	Sample size Mid-therapy	Sample size EOT	Sample size FU	Conclusion
**Cervical cancer**							
Dong et al. 2002 [48]	CSCC	292 (175 invasive cancers)	73		193		No repeated measures. Either collected prior to treatment or after treatment.
Campitelli et al. 2012 [44]	CSCC	16	16	2	2	2	Two cases with recurrence had repeated measurements during palliative treatment. Dynamics in HPVctDNA correlated to outcome.
Cabel et al. 2021 [40]	CC	55	14 (41)	14	14 (25)		Detectable HPVctDNA after treatment was associated with lower DFS: HR = 5.1, *p* = 0.05 Shorter OS: HR = 25.4, *p* < 0.01
Jeannot et al. 2021 [43]	CC	94	94		40	44	Detectable HPVctDNA at end of treatment is associated with recurrence.
**Head and neck cancer**							
Cao et al. 2012 [37]	OPSCC	64	64	14	x	3	For 14 patients HPVctDNA was measured until elimination. Elimination pattern was rapid. HPVctDNA was detectable at the time of relapse in the three investigated patients.
Ahn et al. 2014 [54]	OPSCC	93	35		35		Shorter RFS if EOT pHPV was positive compared to pHPV negative, HR = 12.7
Dahlstrom et al. 2015 [30]	OPSCC	262	262		x	x	ND
Mazurek et al. 2016 [56]	OPSCC	200	28	15	10	5–12	HPVctDNA decreases during therapy.
Lee et al. 2017 [29]	HNSCC	88	47	10	37	10	HPVctDNA after treatment correlates to outcome in a case-based report.
Hanna et al. 2018 [31]	OPSCC (advanced)	22	22	x	x	x	HPVctDNA can capture dynamics in tumor burden and detect early treatment response.
Hanna et al. 2019 [32]	OPSCC (advanced)	21	21	x	x	x	Salivary HPVctDNA mirrors treatment response. Plasma HPVctDNA holds prognostic value.
Chera et al. 2019 [33]	OPSCC	103	103	67	67	67	Rapid HPVctDNA clearance during CRT (week 4) implied a low risk of treatment failure (*p* < 0.01)
Damerla et al. 2019 [66]	OPSCC	97	97	28–(68*)	28–(68)*	28–(68)*	With few exceptions, the HPVctDNA declined rapidly during treatment with complete elimination after 7 weeks.
Chera et al. 2020 [38]	OPSCC	115	86		115	11	Two consecutive post-therapy tests: PPV of HPVctDNA for recurrence = 94% NPV of HPVctDNA for recurrence = 100% Mean lead time to biopsy proven recurrence 3.9 months
Rutkowski et al. 2020 [57]	OPSCC	66	66			66	Detectable HPVctDNA 12 weeks after treatment is strongly associated with later detected recurrence. PPV of HPVctDNA for recurrence = 83 % NPV of HPVctDNA for recurrence = 100%
Reder et al. 2020 [58]	OPSCC	30	28			30	Descriptive Four cases with recurrence all showed increasing HPVctDNA levels prior to clinical diagnosis of recurrence.
Tanaka et al. 2021 [28]	HNSCC	35	30		30		PPV of HPVctDNA at end of treatment for treatment failure = 100% NPV of HPVctDNA at end of treatment for treatment failure = 89.7%
Harring et al 2021 [59]	OPSCC (R/M+)	12	12	x	x	x	Samples were drawn at various time points. 60% increase and an early HPVctDNA progression after first cycle was prognostic for progression by RECIST.
Routman et al. 2022 [62]	OPSCC	45	45 (32)			(32) 159*	Post-op HPVctDNA was associated with recurrence and survival.
Cao et al. 2022 [34]	OPSCC	34	28	x	21	22	Early changes in HPVctDNA were associated with freedom from progression.
Adrian et al. 2023 [35]	OPSCC	136	136		x		Prognostic value of AUC-ctHPV16DNA changes.
Califano et al. 2023 [36]	OPSCC	233	207			204	Post treatment HPVctDNA was associated with recurrence with a median lead time of 19 days, mean 122 days to failure.
**Anal cancer**							
Cabel et al. 2017 [63]	SCAA	1	1				Consecutive samples during immunotherapy mirror response.
Cabel et al. 2018 [25]	SCCA	33	33		18		Most patients eliminate HPVctDNA after CRT. Residual HPVctDNA after CRT is strongly associated with shorter DFS.
Bernard Tessier et al. 2019 [26]	SCCA (metastatic)	59	57		44		Baseline levels were correlated to outcome. Responding patients had lower levels after chemotherapy and the median change correlated to radiologic response. Residual HPVctDNA after chemotherapy was correlated to outcome.
Lee et al. 2020 [65]	SCCA	21	21		18		Case-based description. Potential to predict disease response and recurrence.
Lefevre et al. 2021 [19]	SCCA	88	73	72	64	41	Elimination patterns significantly correlate to outcome, *p* < 0.01
Mazurek et al. 2023 [27]	SCCA	62	35	X	x	x	Molecular detection of HPVctDNA correlated to recurrence.

HPV: human papilloma virus. HPVctDNA: circulating human papilloma virus DNA. FU: follow-up. EOT: end of treatment. CSCC: cervical squamous cell carcinoma. HNSCC: head and neck squamous cell carcinoma. OPSCC: oropharyngeal squamous cell carcinomas. SCCA, squamous cell carcinoma of the anus. x: no information on sample size. ND: Not done. DFS: disease free survival. HR: hazard ratio. RFS: recurrence free survival. CRT: chemoradiotherapy. PPV: positive prognostic value. NPV: negative prognostic value. AUC: area under the curve.

* Metastatic/advanced setting.

**Case-based description ***Retrospective cohort. ****Number unknown, 68 patients with multiple samples, 28 with complete weekly sample sets. *****45 pre-operative, 159 post-operative of which 32 had both samples available.

### HPVctDNA in Head and Neck Squamous Cell Carcinoma

A total of 22 studies comprising more than 1,000 patients were included (mostly OPSCC) and HNSCC is the HPV related SCC with the most data on HPVctDNA.

The sensitivity and specificity in the studies have increased over the years. Recently, Tanaka and colleagues reported sensitivity and specificity > 90% for the measurement of HPV16 [28]. A direct comparison of sensitivity and specificity between studies is difficult due to different cohorts and different methods used. The highest sensitivity was achieved for cases with matching tissue HPV classification compared to cases with matched p16 staining [29]. The number of investigated/detected HPV subtypes varies between studies. Nine studies reported only on HPV16 whereas six studies reported on two HPV subtypes (16 and 18, or 16 and 33). Others investigated a broad panel of subtypes, but the subtype distribution is still not established. A correlation between HPVctDNA and tumor burden was observed (Table 1).

The prognostic value of HPVctDNA prior to treatment was investigated in seven studies. Dahlstrom et al. found no correlation between the pre-treatment level of HPVctDNA and outcome after primary treatment [30]. Studies in metastatic OPSCC found a correlation between the level of HPVctDNA and the site of metastasis [31, 32], and Chera et al. developed a prognostic profile with a combination of the pre-treatment HPVctDNA level and the HPVctDNA elimination pattern during treatment [33]. There was a favorable outcome for patients with a high pre-treatment HPVctDNA level and a fast elimination. Of the three most recent publications, Cao et al. and Adrian et al. suggest a prognostic value of pre-treatment levels, in contrast to data presented by Califano et al. [34–36].

Eighteen studies reported serial HPVctDNA measurements, 15 studies during primary treatment and 3 studies during chemotherapy in metastatic HNSCC (Table 2). Across studies HPVctDNA dynamics correlated to treatment outcome. Clearance of HPVctDNA after primary treatment holds positive prognostic value for tumor control, whereas the presence of, or increase in HPVctDNA after treatment was related to poor outcome and a high risk of failure. However, data were often mentioned in descriptive terms only, or case-based reports and the statistical significance vary [29, 30, 33, 37]. Two studies mention a potential positive lead time from HPVctDNA detection to clinical diagnosis of recurrence [36, 38]. Overall, studies indicate a potential for HPVctDNA measurements, but none compared HPVctDNA monitoring to standard evaluation.

### HPVctDNA in cervical squamous cell carcinoma

In the 1990s pioneering work of HPVctDNA measurement in CCSCs was described [39]. Today, the total number of studies is still limited (14 publications). Five studies include pre-cancer stages, and with few exceptions (2/125 cases) HPVctDNA was solely detectable in patients with invasive carcinomas. Mainly HPV16 and 18 were investigated, but recently Cabel et al. investigated and detected 8 different HPV subtypes. The rarer subtypes comprised around 10% in total [40].

The correlation between HPVctDNA level and tumor stage was described in nine studies, mainly descriptive or with a trend for correlation. The prognostic value of HPVctDNA was previously described already in 2001 [41], where HPVctDNA was detected pre-treatment in 6 of 50 patients. Two were later diagnosed with distant metastases, while the remaining four were already diagnosed with distant disease. None of the patients with HPVctDNA-negative pre-treatment samples experienced distant treatment failure. Later, Cheung et al. detected HPVctDNA in 77 of 138 patients and found an association between high pre-treatment levels and risk of recurrence and death [42].

Cabel and co-workers found a significant correlation between the persistence of detectable HPVctDNA post-treatment and a decline in disease-free survival by analyzing serial measurements in 14 patients during primary treatment [40]. This was supported by the data from Jeannot et al. [43]. Campitelli et al. presented two cases with metastatic disease where HPVctDNA dynamics implied a correlation to outcome [44].

### HPVctDNA in Esophageal Squamous Cell Carcinoma

We identified no studies on HPVctDNA in ESCC. However, one cross-sectional study on EAC patients demonstrated that HPVctDNA detection is possible, with increasing frequency from healthy control (*n* = 49) over BE (*n* = 48) to EAC (*n* = 41) irrespective of viral tissue/tumor status and within the subset of patients with HPV-positive tissue (*n* = 35) [45]. The lack of investigations on HPVctDNA in ESCC probably reflects uncertainty about HPV’s pathogenetic role in this disease.

## HPV subtypes and HPVctDNA detection rate across tumor types

Table 1 and Supplementary Table 1 show the materials analyzed, type of method, and HPV subtypes detected. The most common subtypes are 16 and 18, but more rare subtypes were detected. Some cases of HPVctDNA-positive were tumor p16-negative [19]. Notably, data from CC suggested that p16 staining does not cover subtype 58 [46]. Table 1 also reveals a large variation in diagnostic performance of HPVctDNA but generally high performance of the more recent tests. The data background is not sufficient to perform statistical analysis on the influence of laboratory methods, source of ctDNA detection or tumor site. More recent studies have used a ddPCR platform, with sensitivity reported up to 100%, but Lee et al. used NGS with promising results [29].

## Pre-treatment HPVctDNA measurement and correlation with clinical parameters

Most studies suggest a correlation between baseline HPVctDNA and clinical baseline parameters such as stage. A total of 28 studies measured HPVctDNA in patients with early/small cancers, but the detection rates in these subgroups vary. The data suggest that not only HPVctDNA detection rates but also the higher quantitative levels at baseline seem to correlate with increasing disease stage, in line with studies in other cancers. In contrast, Chera et al. reported lower levels in patients with T3 tumors than in T2 tumors, but the sample size was small [33].

Only a few studies analyzed HPVctDNA levels as a pre-treatment prognostic parameter, and the number of patients included did not allow for multivariate analysis. The independent value of HPVctDNA measurement prior to initiation of curative treatment therefore still needs to be demonstrated.

## Value of repeated HPVctDNA measurements during CRT

Twenty-eight studies included more than a single measurement, the majority with a pre-treatment sample plus end of therapy (EOT) and/or follow-up, whereas only a few analyzed HPVctDNA mid-therapy (Table 2).

Campitelli et al. described two cases with CSCC where HPVctDNA elimination during therapy corresponded to a CR in one patient and unchanged MRI status in another [44]. The two cases demonstrated the value of HPVctDNA as a marker of MRD and HPVctDNA elevation preceding detection of clinical recurrences. Lee and colleagues performed NGS based HPVctDNA detection in OPSCC and demonstrated elimination at EOT, and a single HPVctDNA-positive case at EOT corresponding to treatment failure. Only 10 mid-treatment samples were available in this cohort and the elimination pattern therefore not statistically addressed [29]. Damerla et al. observed heterogeneous kinetics by weekly HPVctDNA measurements during therapy in 28 OPSCC patients, with general decline by EOT [66]. Chera et al. investigated the HPVctDNA clearance profile by weekly measurements in 67 patients with OPSCC and suggested that rapid clearance >95% by the fourth week of CRT was associated with a higher chance of disease control [33]. Lefévre et al. revealed 3 different elimination patterns of HPVctDNA in SCCA with statistical correlation to outcome [19]. All 12 patients with fast elimination at mid-therapy obtained disease control, whereas slow elimination by EOT identified a subgroup of patients with risk of local failure, and patients with persistent HPVctDNA by EOT showed risk of distant failures. It is hypothesized that a slow elimination pattern indicates a high risk of local or distant treatment failure and patients presenting this pattern might benefit from treatment adaption with an EOT boost, intensified chemotherapy, or post-CRT adjuvant systemic treatment. Conversely, patients with a fast tumor DNA elimination during the treatment course, could be candidates for dose reduction and thereby spared from unnecessary toxicity. However, the optimal timepoint for measurement during therapy is not established, and only prospective studies with frequent sampling will validate the utility of HPVctDNA elimination patterns during therapy and subsequently the potential of treatment adaption based on the HPVctDNA kinetics.

## HPVctDNA as a marker of minimal residual disease and early detection of failure

CtDNA is an established marker of MRD in cancers after primary surgery. The risk of recurrence in patients with post-surgical ctDNA is high toward 100% [67]. CtDNA clearance has been demonstrated during post-operative chemotherapy and the ctDNA information is used in clinical trials. With escalation strategies in ctDNA-positive patients to increase the chance of elimination after surgery and conversely de-escalation approaches in ctDNA-negative cases, hereby omitting chemotherapy to avoid unnecessary toxicity. There is an increasing number of prospective clinical trials investigating ctDNA guided post-surgical treatment decisions.

The importance of ctDNA MRD in SCCs was reported by Routman et al. analyzing 32 post-operative samples in OPSCC, confirming shorter recurrence free survival in patients with detectable post-operative HPVctDNA [62].

In contrast to the immediate effect of surgery, the final biological elimination of tumor cells after radiotherapy varies greatly up to several months after primary CRT, and the optimal time point for final response evaluation is still undefined in most SCCs. The elimination time of ctDNA is short between minutes and a few hours and it is therefore highly relevant to analyze the clearance of HPVctDNA after CRT in SCCs. HPVctDNA detection could potentially add to establish time points for final response evaluations after CRT and thereby aid in decisions on salvage surgery. Structured repeated measurements after CRT for SCC have however not been presented yet.

HPVctDNA positivity in an early post-treatment sample after end of CRT seems to imply a poor prognosis. In SCCA, all patients (3/18) with HPVctDNA detected <30 days post CRT experienced recurrence compared to only one of the ctDNA-negative patients [25]. Tanaka et al. reported that the post-treatment (10–12 weeks post CRT) HPVctDNA level in 30 patients treated for OPSCC was significantly higher in patients with treatment failure compared to patients who did not recur. Combining the HPVctDNA results with PET-CT metabolic response could add further prognostic information [28]. Rutkowski and colleagues presented that HPVctDNA 12 weeks after treatment was strongly correlated to recurrence [57]. Mazurek et al. reported on a single patient with HPVctDNA recurrence, who was treated with chemotherapy and achieved HPVctDNA elimination and subsequent long-term survival [27]. HPVctDNA testing could potentially classify patients into more nuanced high or low-risk groups during follow-up and allow for early detection of recurrences. Another aspect is the lead time between the HPVctDNA detected recurrence/MRD and the clinically observed failure. Mean lead times of 3.9 months and 122 days have been reported in OPSCC [36,38] and thus a clear clinical relevance.

Table 2 shows studies with repeated measurements, including samples drawn during follow-up. In general, a positive sample at any timepoint post CRT confirms a risk of treatment failure or recurrence, but statistical evaluation of positive predictive and negative predictive values is naturally hampered by the exploratory nature of the studies, the high response rates in most SCCs and consequently the low number of events. However, the signal remains strong, necessitating adequately powered studies to determine the optimal time points for clinical and ctDNA-based response evaluation and to establish the clinical utility of ctDNA as a recurrence marker compared to current clinical standards.

## Clinical value of HPVctDNA in metastatic settings

Metastatic SCC is rare and the number of studies having investigated HPVctDNA limited. In other cancers, multiple studies show a clear prognostic value of pre-treatment ctDNA levels, and the clinical utility during systemic treatment wide explored [68, 69]. In general, ctDNA response both mirrors the clinical response and shows potential as a better surrogate endpoint in the metastatic setting than standard assessments [70]. In SCC data are limited. Bernard-Tessier et al. presented results in anal cancer, treated according to the Epitopes trial, and reported that higher pre-treatment HPVctDNA levels were associated with more advanced disease, that HPVctDNA declines with response and that post-therapy HPVctDNA status was significantly associated with PFS and OS [26]. Hanna et al. presented data from a small cohort, indicating that HPVctDNA captures dynamics in tumor burden [31]. Haring et al. presented results from 12 patients which indicated that increasing levels above 60% at the time of re-imaging was associated with progression, and that early HPVctDNA changes between the first two cycles seem to correlate with clinical outcome [59]. These results are in line with emerging data on other diseases. Studies are needed to establish relevant definitions for HPVctDNA response and progression, and the clinical utility compared to standard evaluation tools [71].

## Gaps of knowledge and design of observational prospective studies

### HPVctDNA in surgically treated patients

In some diseases, decisions between surgery or definitive CRT are a multidisciplinary challenge, and studies designed to investigate the utility of HPVctDNA as a tool for pre-treatment risk assessment should be considered. This implies a blood sample drawn prior to surgery, correlation to imaging results and pathology, and relevant correlation to recurrence, DFS and OS endpoints.

Since the optimal time point for post-surgical sampling is not defined, valuable information will be retrieved from repeated post-surgical sampling to identify, which post-surgical stress responses and total DNA level peaks [71]. Early assessment will allow for selection to adjuvant therapy in high-risk patients with HPVctDNA MRD, later for early detection and treatment of recurrences. To investigate the HPVctDNA lead time potential, pairwise HPVctDNA analysis and clinical/imaging procedures are essential.

### Utility of HPVctDNA in patients treated with CRT

Definitive CRT poses a risk of severe acute and late morbidity. HPVctDNA should be investigated as a potential tool for improved pre-treatment risk assessment, investigating results in relation to pre-treatment clinical information and imaging, and potentially to radiotherapy treatment plans. Well-designed prospective studies of the prognostic value of pre-treatment HPVctDNA levels are essential for statistically powered evaluations of HPVctDNA as a single parameter. The aim is to refine risk categories beyond the current TNM classification. This approach should guide future clinical trials exploring dose escalation or de-escalation strategies.

**Figure 2:**
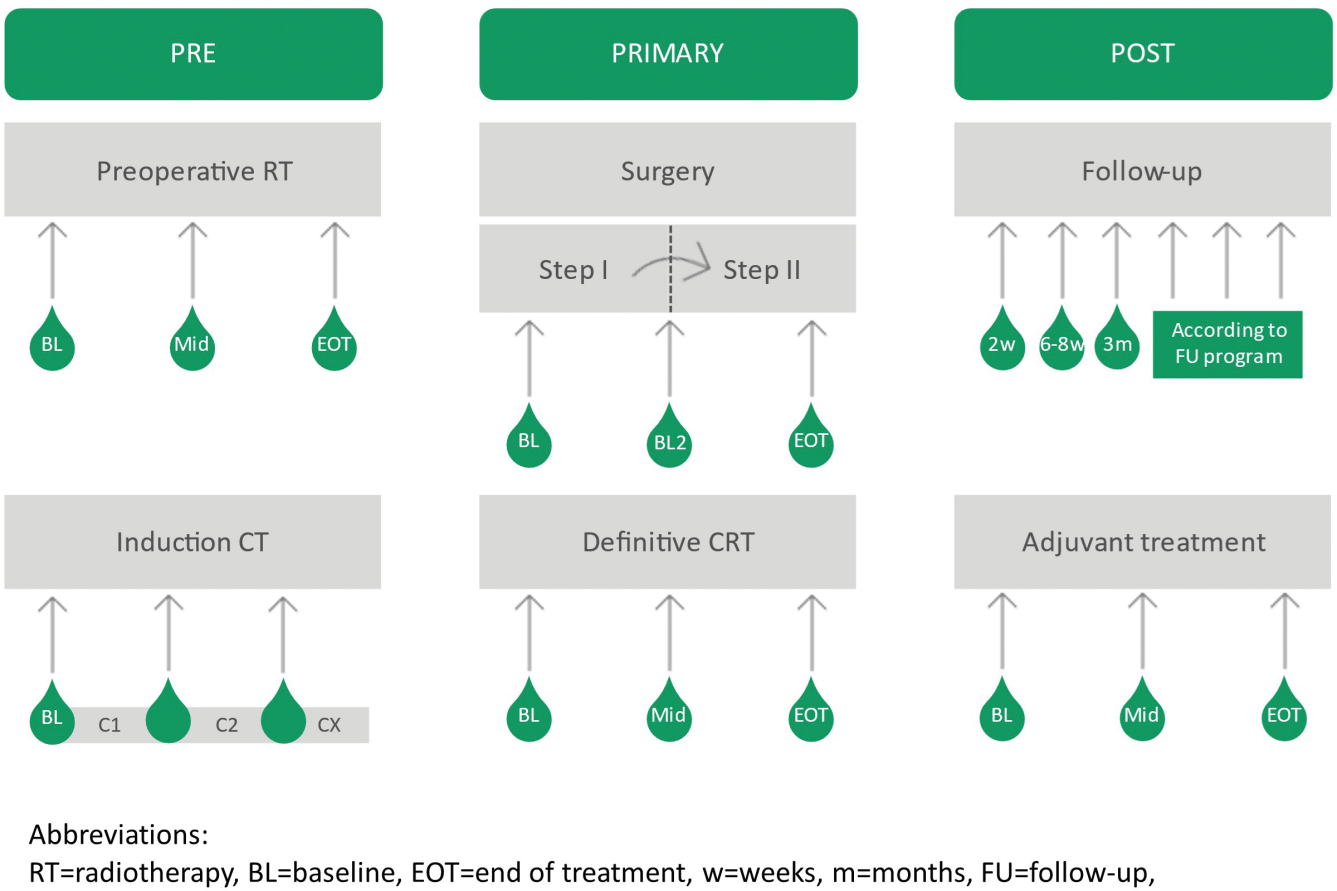
Suggested timepoints for sampling in future observational studies in primary surgery, chemoradiotherapy and during induction or adjuvant chemotherapy. Each sampling timepoint has a potential clinical relevance such as adding to the selection of primary surgery or CRT, treatment adaption based on sampling during CRT or CT, decisions on additional adjuvant chemo or radiotherapy, and detection of minimal residual disease after curative therapy.

Repeated measurement during CRT can allow for mid-treatment reassessment of the therapy and for adaptation during the course. Optimal timing for early adaptation is to be defined, thus repeated measurements during therapy are essential (Figure 2). Sampling within the last week of CRT can inform decisions on adding an EOT boost or intensifying chemotherapy in slow elimination cases. The biological tumor reduction rate post-CRT is uncertain, so studying HPVctDNA elimination could aid in defining the optimal timing of final response evaluation. Repeated measurements after EOT will be important, especially if correlated with clinical and imaging procedures. Follow-up sampling should be combined with clinical/imaging status to enable early detection of recurrences and to assess the lead time between HPVctDNA detected recurrence and clinical relapse signs.

### HPVctDNA measurements during systemic treatment for advanced disease

A pre-treatment prognostic factor could add to clinical information and treatment decisions, particularly in the advanced setting with poor prognosis and limited efficacy from available systemic options. Recent findings suggest that ctDNA response might serve as a more reliable surrogate for OS compared to RECIST evaluations. Thus, assessing HPVctDNA response at the first evaluation of treatment response is essential. Conversely, early HPVctDNA progression may signal poor prognosis and a lack of benefit from systemic treatment, making sampling before the first three cycles relevant. Sampling at the time of progression could identify new targets for precision medicine, bringing value to future research in this area.

## Clinically relevant designs for HPVctDNA guided studies

At current time there is strong data supporting the use of ctDNA as a marker of MRD across tumor types. ctDNA information is used to escalate or deescalate post-surgical adjuvant systemic therapy and for adding information to follow-up. Whereas escalation strategies in ctDNA-positive patients seem straightforward in most settings, the controversial points are feasibility of randomization, use of additional advanced imaging for example PET-CT scans, how to de-escalate and the primary endpoint. Some studies are designed with a strong endpoint such as recurrence or OS, whereas ctDNA clearance is increasingly used as primary endpoint. Studies that address ctDNA as replacement or substitute to imaging procedures are awaited.

Similar studies can be designed in SCCs, but to allow for ctDNA guided treatment decisions, it is essential that the method for HPVctDNA analysis has undergone pre-analytical and analytical validation, provides high sensitivity and specificity, and is feasible in low total DNA samples. In the MRD situations, binary reliable detection is needed. Finally, the assays must include both multiple relevant HPV subtypes and prove high feasibility in terms of fast laboratory results.

Less data allows for prospective studies of adaptation treatment, where reliable quantitative measures are needed. Validation of the observations from the current literature must be confirmed before entering prospective clinical intervention trials based on HPVctDNA results during treatment.

## Conclusions

Strong data support the use of ctDNA as a marker of MRD across tumor types and potential as tool for adjuvant treatment guidance and follow-up. Similar studies can be designed in SCCs. Limited data warrants prospective studies of primary or palliative treatment adaption, and validation of the existing findings is essential before initiating clinical intervention trials based on HPVctDNA results during treatment. The HPVctDNA analysis method requires pre-analytical and analytical validation to ensure high sensitivity and specificity and feasibility in low DNA samples. It must include multiple relevant HPV subtypes and offer fast laboratory results while remaining feasible.

In conclusion, also in SCC data is emerging to confirm the major clinical potential of ctDNA measurement for risk assessment and treatment monitoring. Structured panSCC evaluations should be considered to allow for relevant sample sizes for statistical validation and to identify potential differences between the sub entities.

## Supplementary Material

The clinical utility of circulating human papillomavirus across squamous cell carcinomas

## Data Availability

No new data were created or analysed during this study. Data sharing is not applicable to this article.
