# Supplementary material for: The clinical utility of circulating human papillomavirus across squamous cell carcinomas
Source: Acta Oncol. 2025 Jan 2;64:41288. doi: 10.2340/1651-226X.2025.41288 (PMC11711493; doi:10.2340/1651-226X.2025.41288)
Supplement: The clinical utility of circulating human papillomavirus across squamous cell carcinomas [file AO-64-41288-s1.pdf]

Supplementary material has been published as submitted. It has not been copyedited, or typeset by Acta Oncologica

Supplementary Table 1 for the manuscript "The role of circulating HPV in SCC – From hypothesis generating studies towards designing prospective circulating HPV guided trials"

Studies investigating multiple HPV subtypes		
**Reference**	**Investigated subtypes**	**Detected subtypes**
Cabel et al. 2021 [40]	16, 18, 31, 33, 35, 45, 52, 58, 73	16, 18, 31, 33, 35, 45, 52, 58, 73
Hanna et al 2018 [31]	16, 18, 31, 33, 45	NA
Hanna et al 2019 [32]	16, 18, 31, 33, 45	NA
Chera et al. 2019 [33]	16, 18, 31, 33, 35	16, 31, 33, 35
Chera et al. 2020 [38]	16, 18, 31, 33, 35	NA
Siravengna et al. 2020 [67]	16, 18, 33, 35, 45	16, 35, 45
Routman et al. 2022 [49]	16, 18, 31, 33, 35	16, 18, 33, 35
Adrian et al. 2023[35]	16 + 40 subtypes	6, 11, 16, 18, 31, 33, 35, 35.6624A, 82, 90
Lee et al. 2020 [71]	16, 18, 31, 33, 35, 45, 52, 58	NA
Lefevre et al. 2021 [19]	16, 18, 31, 33, 51, 58	18, 31, 33, 51, 58

Supplementary Table 1. Subtypes of HPV investigated and detected in studies analysing multiple subtypes.
